# MIA-Clustering: a novel method for segmentation of paleontological material

**DOI:** 10.7717/peerj.4374

**Published:** 2018-02-23

**Authors:** Christopher J. Dunmore, Gert Wollny, Matthew M. Skinner

**Affiliations:** 1 School of Anthropology and Conservation, University of Kent, Canterbury, Kent, UK; 2 Department of Human Evolution, Max Planck Institute for Evolutionary Anthropology, Leipzig, Germany

**Keywords:** Digital image processing, Micro-CT, Machine-learning, Fossil, Trabecular bone

## Abstract

Paleontological research increasingly uses high-resolution micro-computed tomography (μCT) to study the inner architecture of modern and fossil bone material to answer important questions regarding vertebrate evolution. This non-destructive method allows for the measurement of otherwise inaccessible morphology. Digital measurement is predicated on the accurate segmentation of modern or fossilized bone from other structures imaged in μCT scans, as errors in segmentation can result in inaccurate calculations of structural parameters. Several approaches to image segmentation have been proposed with varying degrees of automation, ranging from completely manual segmentation, to the selection of input parameters required for computational algorithms. Many of these segmentation algorithms provide speed and reproducibility at the cost of flexibility that manual segmentation provides. In particular, the segmentation of modern and fossil bone in the presence of materials such as desiccated soft tissue, soil matrix or precipitated crystalline material can be difficult. Here we present a free open-source segmentation algorithm application capable of segmenting modern and fossil bone, which also reduces subjective user decisions to a minimum. We compare the effectiveness of this algorithm with another leading method by using both to measure the parameters of a known dimension reference object, as well as to segment an example problematic fossil scan. The results demonstrate that the medical image analysis-clustering method produces accurate segmentations and offers more flexibility than those of equivalent precision. Its free availability, flexibility to deal with non-bone inclusions and limited need for user input give it broad applicability in anthropological, anatomical, and paleontological contexts.

## Introduction

Over the last decade there has been an abundance of high-resolution micro-computed tomography (μCT) studies within the paleontological and anthropological communities, likely due to the ability of this method to non-destructively image extant and fossil specimens. This has been used to investigate the inner osseous architecture of a diverse range of orders including: primates (Ryan et al., 2010), galliformes (Pontzer et al., 2006), xenarthrans (Amson et al., 2017) and diprotodontians (Biewener et al., 1996). The technique allows the visualization of internal structures, such as trabeculae (Fajardo et al., 2007), the enamel–dentine junction of teeth (Skinner et al., 2009) or the inner ear (Spoor et al., 2007). This is of particular importance for fossils, whose inner architecture could only be destructively analyzed otherwise (Witmer et al., 2008; Kivell, 2016). To visualize very small biological structures, it is necessary to ensure adequate X-ray penetration of the bone or fossil material being CT-scanned, as well as to control for common artifacts such as beam hardening (Herman, 1979). To digitally measure these structures and their properties, it is necessary to define them in the scan image and so the image must be accurately segmented (Hara et al., 2002).

Various segmentation protocols have been developed for anthropological applications. Simple thresholding involves the visual selection of a grayscale value, any part of the image composed of voxels above this value is considered the phase of interest. Iterative adaptive thresholding (Ridler & Calvard, 1978; Trussell, 1979; Ryan & Ketcham, 2002) improves on this simple thresholding by optimizing the threshold value between the present phases. Conversely, half-maximum-height thresholding (Spoor, Zonneveld & Macho, 1993; Coleman & Colbert, 2007) recalculates the threshold over a row of pixels, which cross a phase boundary, periodically in the *z*-axis of a three-dimensional (3D) image. These three methods are all sensitive to intensity inhomogeneity and background noise in a scan (Scherf & Tilgner, 2009). In all cases, a grayscale value threshold calculated from a different or larger section of an image may not accurately segment all parts of the structure.

Instead of using grayscale values alone, region-based segmentation approaches incorporate the spatial information in a scan. Region growing methods use seed points, manually selected by the researcher, known to be in the phase of interest. A segmented region is then grown from the seed by connecting neighboring voxels that meet specific, pre-defined criteria (Pham, Xu & Prince, 2000). Region splitting, conversely, does not use seed points but divides the image into distinct regions and refuses the image based on selected criteria. Both region-based approaches, however, often require a priori knowledge of image features to select seed points or criteria, and can be sensitive to intensity inhomogeneity (Pham, Xu & Prince, 2000; Dhanachandra & Chanu, 2017).

Edge-detection-based segmentation offers an alternative method that discerns the transition between two phases and delineates these voxels as an edge. The Ray Casting Algorithm (RCA, Scherf & Tilgner, 2009) is an example of this method used in anthropology (Tsegai et al., 2013). This algorithm uses a 3D-Sobel filter to mark voxels at the peak of rapid changes in grayscale values and subsequently removes the rest of the image with a non-maximum suppression filter. To be considered part of the remaining edge of the phase of interest, the gradient of the grayscale transition must be above a user-defined “minimum edge strength” parameter. This one-voxel-thick edge may have infrequent gaps due to local, more gradual, transitions not quite satisfying the “minimum edge strength” threshold. In order to ameliorate this, a series of rays are subsequently cast at 11.25° steps around the normal of each edge voxel in an arc of ±45°. The rays are set to terminate on meeting a voxel with the specified “minimum edge strength,” so edge voxels that neighbor these gaps terminate the rays at most angles, and the gap is closed. The RCA segmentation produces a structure with the continuous edge described (Tsegai et al., 2013).

Edge-based segmentation techniques provide an advantage over other techniques in that they are resistant to the effects of both background noise and intensity inhomogeneity. Tests of segmentation methods have found RCA is more accurate than thresholding methods (Scherf & Tilgner, 2009). Similarly, algorithms such as RCA require less prior knowledge of the image, as they need no seed points or initial manual segmentation. Still, the RCA requires the selection of the “minimum edge strength” value and may also incorporate minimum or maximum threshold values. These input values are found during trial segmentation of a sub-set of the data (Scherf & Tilgner, 2009). The selection of these three parameters is partially subjective, as is the case with all segmentation algorithms. This input parameter selection represents another source of error, that an algorithm must be robust to, in addition to background noise and intensity inhomogeneity. An algorithm run with extreme parameters is unlikely to produce an accurate segmentation. With RCA, the same segmentation can be produced with different sets of input values. This equifinality is not a problem of the method per se, but allows for additional potential difficulty in reproducing the same segmentation. A researcher cannot be sure that a visually similar segmentation was produced using the same RCA parameters. Here we present a segmentation method, medical image analysis (MIA)-Clustering, implemented as free- and open-source software (Wollny et al., 2013), that reduces subjective user decisions to a minimum. Broadly, clustering approaches sort the voxels or pixels of an image into a number of clusters defined by the user. This sorting is accomplished by iteratively calculating the center of a cluster and its distance to the other voxels in that cluster. This iteration then converges on stable clusters by minimizing this distance and the voxels in each cluster are segmented as distinct phases. The MIA-Clustering algorithm performs this sorting both globally and locally to segment an image based on its properties.

We test the efficacy of the MIA-Clustering algorithm by segmenting a reference model of known thickness. Results of this segmentation and a RCA segmentation of the same material following Scherf & Tilgner (2009) are compared. To assess the robustcity of the MIA-Clustering algorithm to variation in parameter selection, segmentations of this synthetic material, produced by a range of inputs, are analyzed. Similarly, a fossil sample is segmented with different parameters to assess their effect on the segmentation of a highly variable, embedded, natural structure. This fossil also presents a challenging segmentation, due to multiple phases of invasive matrix as well as bright inclusions, and so permits an assessment of the MIA-Clustering algorithm’s robusticity to background noise and intensity inhomogeneity. The fossil is also segmented using the RCA to compare the simplicity and accuracy of both methods.

## Materials

A coiled stainless steel wire, which is rectangular in cross-section, was used as a reference object of known thickness (40 μm). This materially homogeneous phantom was scanned in air, with the SkyScan 1173 μCT scanner at the Max Planck Institute, Leipzig at 80 kV and 62 μA. This shape of object has previously been shown to both approximate trabecular bone and be susceptible to beam hardening due to its structure (Scherf & Tilgner, 2009). The 4,224 × 4,224 × 2,240 voxel reconstructed image had an isometric voxel size of 7.86 μm. This was cropped to an image size of 3,240 × 3,240 × 150 voxels to reduce processing time. The example fossil was scanned at 90 kV and 200 μA using a Nikon Metrology XTH 225/320 at the University of the Witwatersrand. Permission to use this material was granted by Fossil Access Committee of the Evolutionary Studies Institute at this institution. The reconstructed image was 726 × 551 × 1,826 voxels and had an isometric voxel size of 22.6 μm.

## Methods

### MIA-Clustering algorithm

**Figure 1:**
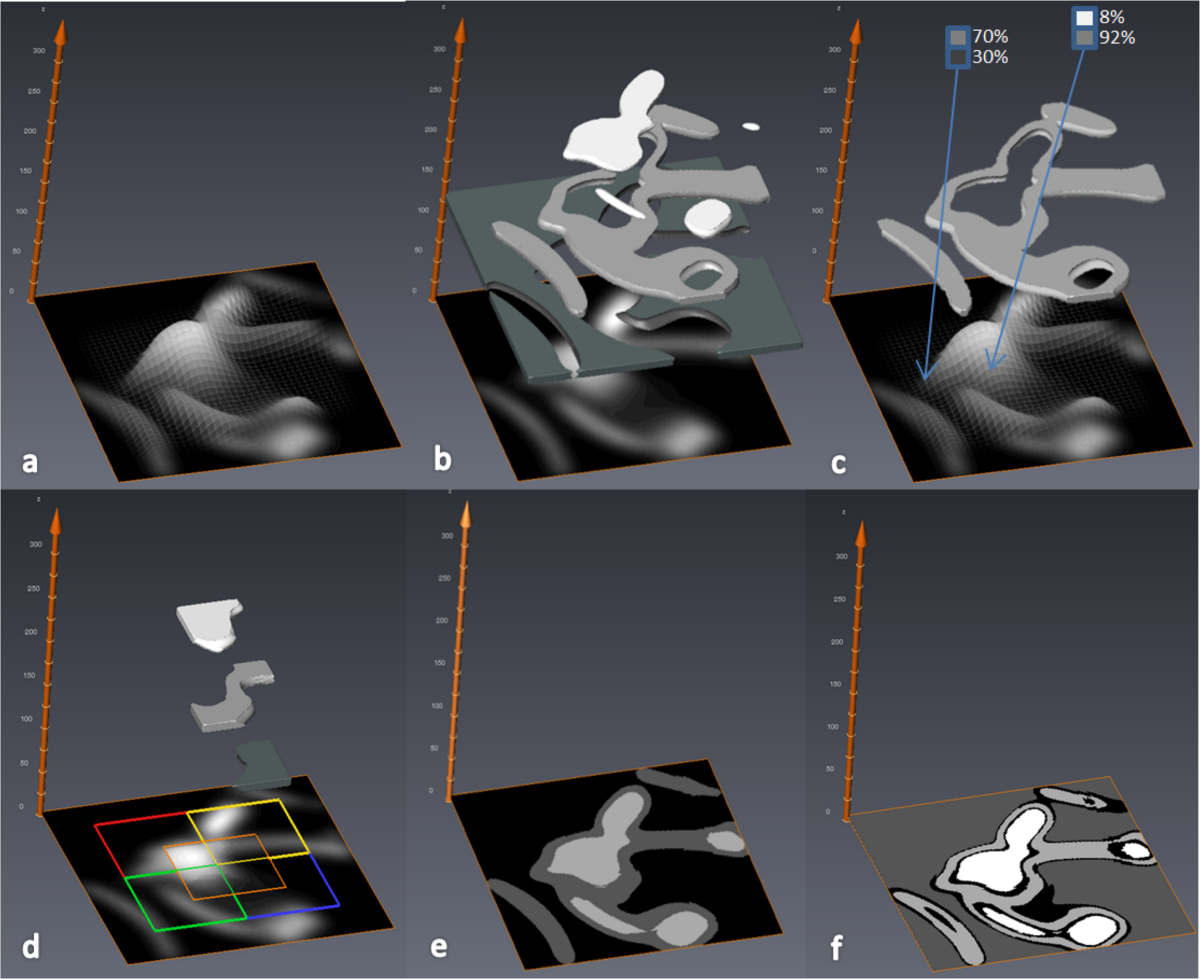
Diagram of MIA-Clustering algorithm in a 2D-image. (A) Gray values are mapped to the *z*-axis. (B) Gray values are initially clustered into three classes by the *K*-means algorithm; the black-class is represented as dark-gray in the 3D overlay for clarity. (C) The fuzzy *c*-means algorithm iteratively estimates a class membership probability vector for each voxel (two example voxels are shown in blue boxes) and globally clusters each voxel based on its highest class probability. (D) Local fuzzy *c*-means clustering is performed in overlapping sub-volumes, here represented by the colored squares. (E) Overlapping class probabilities are merged and voxels are clustered based on their highest membership probability. (F) An optional probability threshold is then applied at an arbitrary 75%, for illustrative purposes. All voxels with their highest membership probabilities below 75% are labeled as zero, or black, and voxels above this threshold are clustered into three classes labeled by gray values elevated by one; here one to three.

The MIA-Clustering algorithm is a machine-learning approach, based on fuzzy *c*-means clustering (Pham & Prince, 1999) and initialized by the *K*-means algorithm (Forgy, 1965; Lloyd, 1982). First, the *K*-means algorithm clusters the input data, based on voxel intensity, into the number of classes specified by the user (Figs. 1A and 1B). A subsequent fuzzy *c*-means algorithm iteratively estimates all class membership probabilities for each voxel, expressed as a vector (Fig. 1C). Based on their highest membership probability, voxels are globally clustered into distinct classes representing structures in the whole image. However, this global segmentation does not always capture fine detail because the input images may suffer from intensity inhomogeneities, which result from scanning artifacts or different levels of fossil mineralization. Therefore, subsequent local fuzzy *c*-means segmentation is applied.

**Figure 2:**
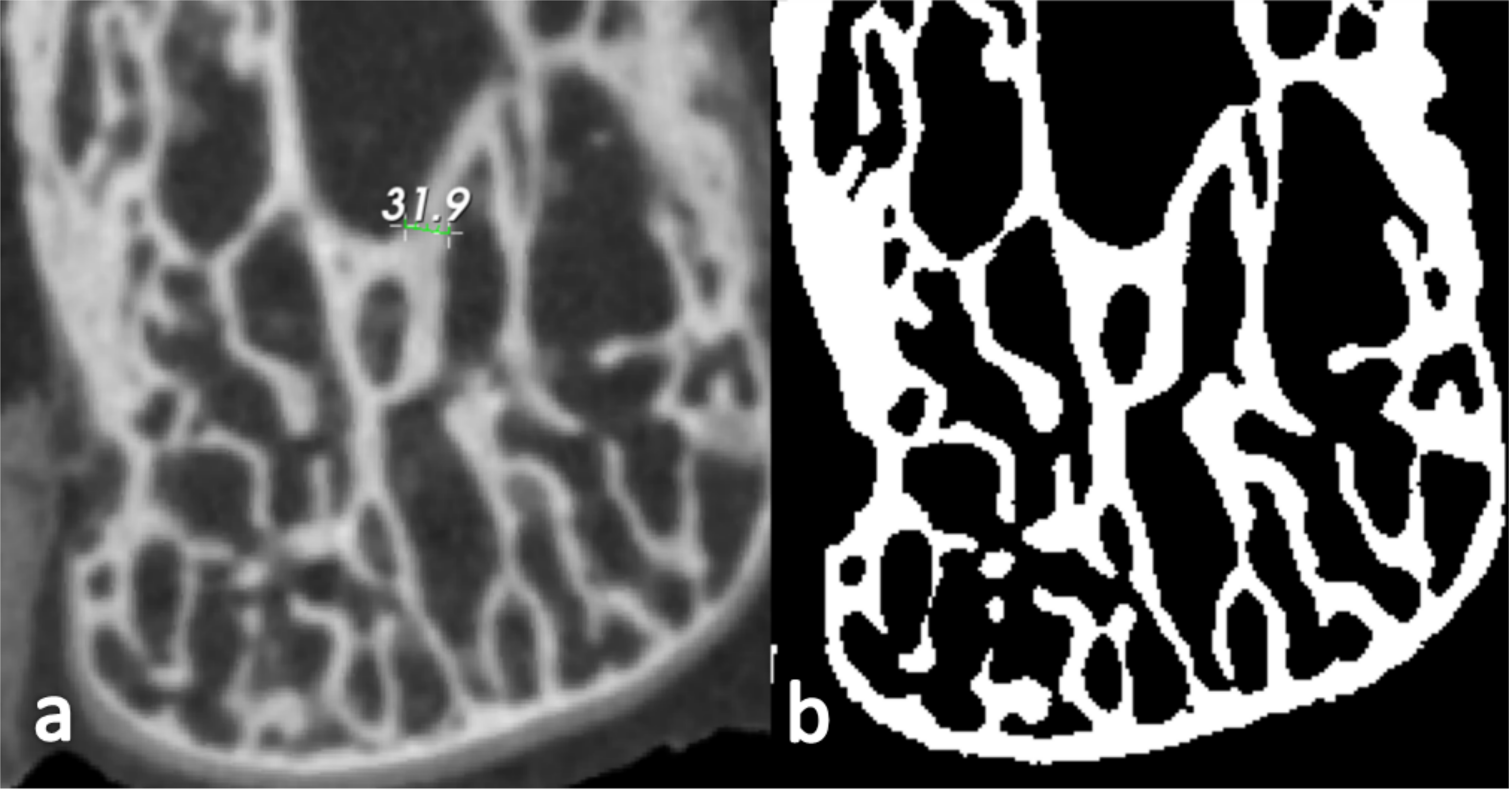
A 2D cross-section image of an example dry bone. (A) One of its thickest trabecular struts in the image measured at ∼32 pixels. (B) A binarized image of the same cross-section after 3D segmentation of the bone, using the MIA-Clustering algorithm. The grid size input parameter selected for the segmentation was 35 voxels as this was just larger than the measurement in (A).

Based on a user-defined grid-size parameter, the volume is subdivided into overlapping cubes. For each cube, the class membership probability vector is initialized by using the globally obtained probabilities (Fig. 1D). If the sum of membership probabilities of all voxels in a sub-volume falls below a threshold, then this class is not taken into account for the local, refined *c*-means clustering. This threshold can be specified by the user if desired, but the default value of 2% appears to generate acceptable segmentations and was used in all cases here. Therefore in this case, if there was no more than 2% of a cube that was globally clustered as a certain class, this class was not considered for that cube’s local *c*-means segmentation. Subsequently, class probabilities for each voxel in overlapping cubes are merged and voxels are assigned to the class for which they have the highest membership probability, producing the whole segmented image (Fig. 1E). This local segmentation allows the algorithm to adapt to local intensity variations. It follows that a grid-size value smaller than the structure of interest will cause the algorithm to attempt to find clusters within these structures, such as small inhomogeneities in cortical bone, that are generally not of interest. Therefore, to balance between adapting to inhomogeneities resulting from imaging artifacts and ignoring small inhomogeneities within the structures of interest, the grid-size parameter selected should be slightly larger than the largest dimension of the phase of interest for the segmentation. For a variable and continuous structure, such as trabecular bone, we recommend looking at two-dimensional (2D) cross-sections in each plane and measuring thicker trabeculae to ascertain their width in pixels. The grid-size value should then be set a few voxels larger than these measurements to ensure the local segmentation is not looking for features within the phase of interest. (e.g., Fig. 2). The global and local segmentations can be generated at the same time for comparison of each segmentation step.

Finally, an optional threshold can then also be applied to the calculated class membership probabilities of each voxel. A voxel is excluded from a class if its highest membership coefficient does not meet or exceed the threshold given. Voxels that do not meet the threshold for their highest class are assigned to a grayscale value of zero and all other classes are elevated by one gray value. Since the vector of membership probabilities sums to one, in practice, this allows the user 50 threshold values (51–100%) to fine tune the segmentation based on the initial, data-led, analysis. The black or zero-class voxels that did not meet the threshold can be considered a margin of error for the segmentation (Fig. 1F).

### Wire segmentation

**Figure 3:**
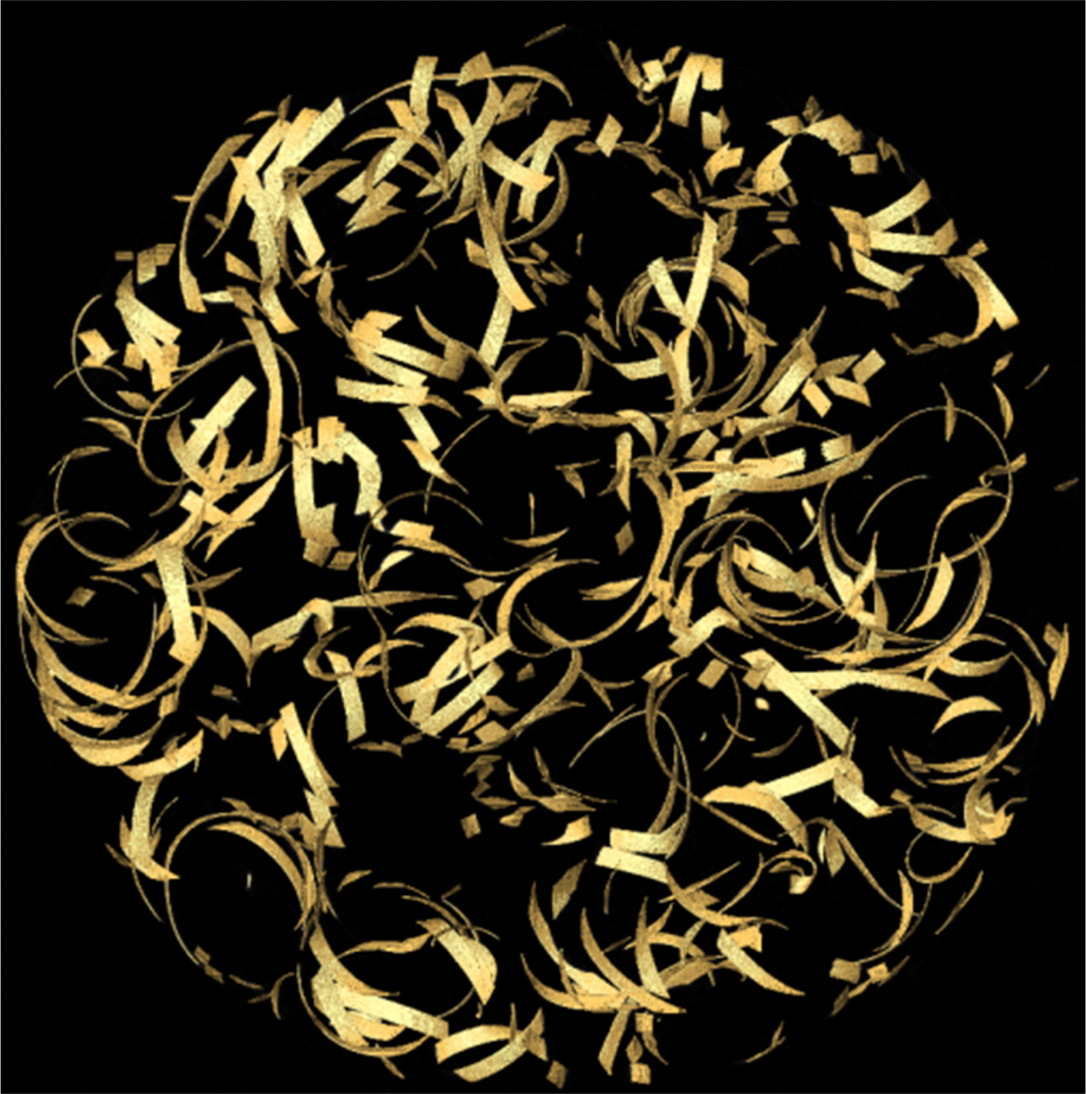
A 3D-surface view of the machined wire phantom.

In order to test its efficacy, the MIA-Clustering algorithm was used to segment a scan of a machined wire phantom, previously measured at 40 μm thickness, following Scherf & Tilgner (2009; Fig. 3). The RCA was also used to segment the same image for comparison. 3D-thickness was measured at every point, in each segmentation of the same 3,240 × 3,240 × 150 voxel volume in the center of the wire, using the BoneJ plugin for ImageJ (Hildebrand & Rüegsegger, 1997; Doube et al., 2010). Average 3D-thicknesses within one voxel, or ∼8 μm, of the measured thickness were considered effective segmentations. In the case of RCA, 3,240 × 3,240 × 10 voxel trial segmentations were run to find the three input parameters that produced acceptable segmentations. In the case of the MIA-Clustering algorithm, the wire thickness of 40 μm divided by the resolution yielded a voxel size of approximately five, thus the grid size was set just above this at seven. The probability threshold used was found after two trial segmentations.

### Parameter robusticity

In order to test the robusticity of MIA-Clustering algorithm, the full range of both input parameters was independently varied and average thickness of the wire in the resulting segmentations was measured. The probability threshold was varied in 5% increments from 50% to 95%. Grid size was varied from the smallest maximum dimension of the dataset, here 150 voxels, to the minimum value of three. The fossil specimen was segmented at grid sizes from 10 to100 voxels, since these more extreme values did not produce a visually satisfactory segmentation. This allowed comparison between segmentations produced by a range of possible values and the grid-size value attained from a cursory visual inspection (e.g., Fig. 2), in a variable structure of largely unknown thickness.

### Fossil application

In order to assess the performance of the presented method on paleontological material, the fossil is segmented using the RCA as well as the MIA-Clustering algorithm; pre- and post-processing steps are described. Every fossil scan is likely to present different issues, owing to disparate diagenetic processes over varying timescales. In some fossils, invasive matrix may be relatively uniform, but overlap in attenuation intensity with the fossil bone phase preventing its removal by a global threshold. Similarly, small bright mineral inclusions may provide grayscale value outliers, thus decreasing contrast in the majority of the material, markedly affecting segmentation approaches based on thresholding of a grayscale value range such as the iterative, adaptive threshold method (Ryan & Ketcham, 2002; Fajardo et al., 2007). Also, cracks and multiple phases of invasive matrix may create edges within the fossil that are distinct from the fossil bone. The present fossil scan contains all of these issues to some extent, as well as a global gradient that becomes brighter towards the center of the fossil. This centrally higher attenuation artifact is the result of photons with less energy than is required to uniformly penetrate this dense fossil and is essentially the inverse of beam hardening.

### Implementation

The RCA segmentations were run as a stand-alone executable on the Windows command line. The MIA-Clustering algorithm was run as command line tool using MIA (Wollny et al., 2013). MIA was run from a Docker image as a Docker container in order to run a lightweight virtual Linux machine in Windows (Boettiger, 2015). This approach allows MIA to be run on most widely available operating systems. Instructions for downloading and use of MIA are available at http://mia.sourceforge.net/.

## Results

### Wire segmentation

Two acceptable sets of parameters were found for RCA segmentations, after at least 10 trial segmentations for each. The probability threshold value for the MIA-Clustering algorithm was found after two trial segmentations at 80% and 90%. MIA-Clustering algorithm segmentations of the 3 gigabyte wire phantom scan ran in ∼10 min using four cores whereas RCA ran this object in ∼8 min using 16 cores.

**Figure 4:**
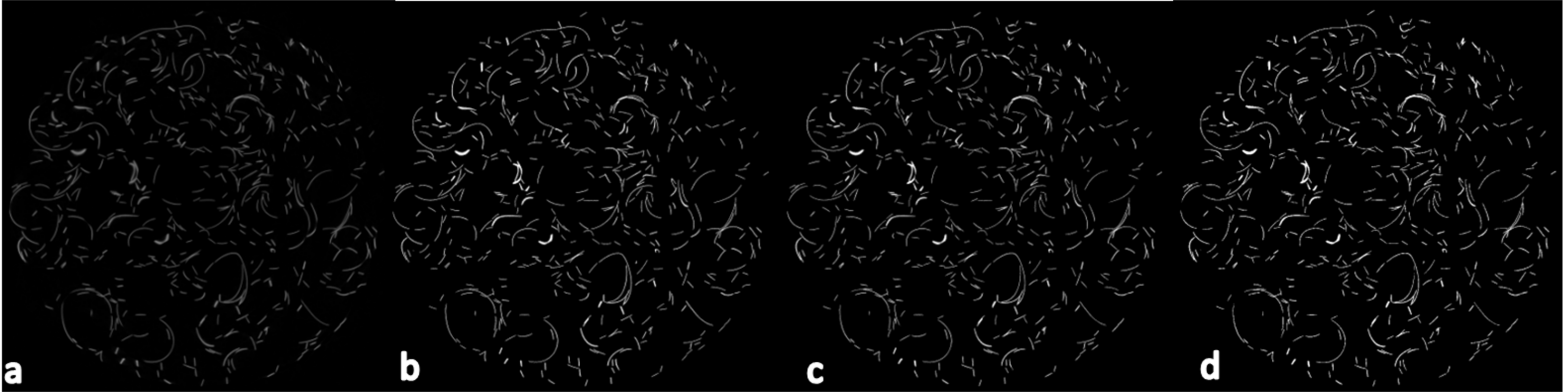
The mid-slice of the wire scan in superior view. (A) The reconstructed image. (B) The segmented image produced by the MIA-Clustering algorithm. (C) The segmented image produced by the RCA.1 and (D) the equifinal RCA.2 segmentation. Note the similarity of the segmentations of (A) in each method (B–D).

**Figure 5:**
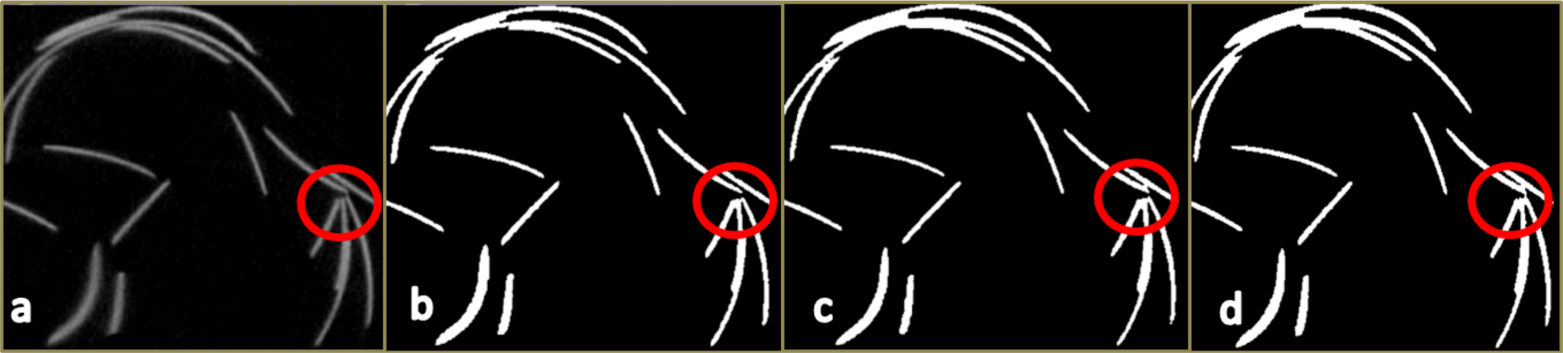
A magnified section of the mid-slice of the wire phantom scan (Fig. 4) in superior view. (A) The reconstructed image. (B) The segmented image produced by the MIA-Clustering algorithm. (C) The segmentation produced by the RCA.1 and (D) the equifinal RCA.2 segmentation. Note the separation of closely packed wire in the red circles in (A) and (B) but not in (C) and (D).

As can be seen in Table 1 and Fig. 4, both algorithms can produce accurate segmentations, segmenting the wire at thicknesses within 1 μm of the known width of the wire. Figure 5, however, demonstrates that at least for some local areas the MIA-Clustering algorithm segments the closely packed, fine structures more accurately than either of the equifinal RCA segmentations. The average thickness values are within 1% and 0.5% of the known thickness, respectively. This is considered acceptable given an isometric voxel size of 8 μm (Table 1). The standard deviation of the thickness measured in the RCA segmentation is slightly higher than the voxel size whereas the MIA-Clustering algorithm segmentation standard deviation is below this level of variability and therefore is the result of partial volume effects.

**Table 1:** Mean and standard deviation of thickness calculated for each segmentation method.

Segmentation method	Thickness mean (pixels)	σ (pixels)	Thickness mean (μm)	σ (μm)
RCA.1	5.054	1.340	39.728	10.533
RCA.2	5.026	1.386	39.508	10.895
MIA-Clustering algorithm	5.111	0.952	40.176	7.484

**Notes:**

RCA.1 used parameters lower threshold: 7,000, upper threshold: 20,000 and minimum edge strength: 5,000; RCA.2 used lower threshold: 18,000, upper threshold: 26,000 and minimum edge strength: 20,000. Note the near identical measurements using two different sets of values. Parameters for the MIA-Clustering algorithm were grid size: 7 and probability threshold 85%.

### Parameter robusticisty

**Figure 6:**
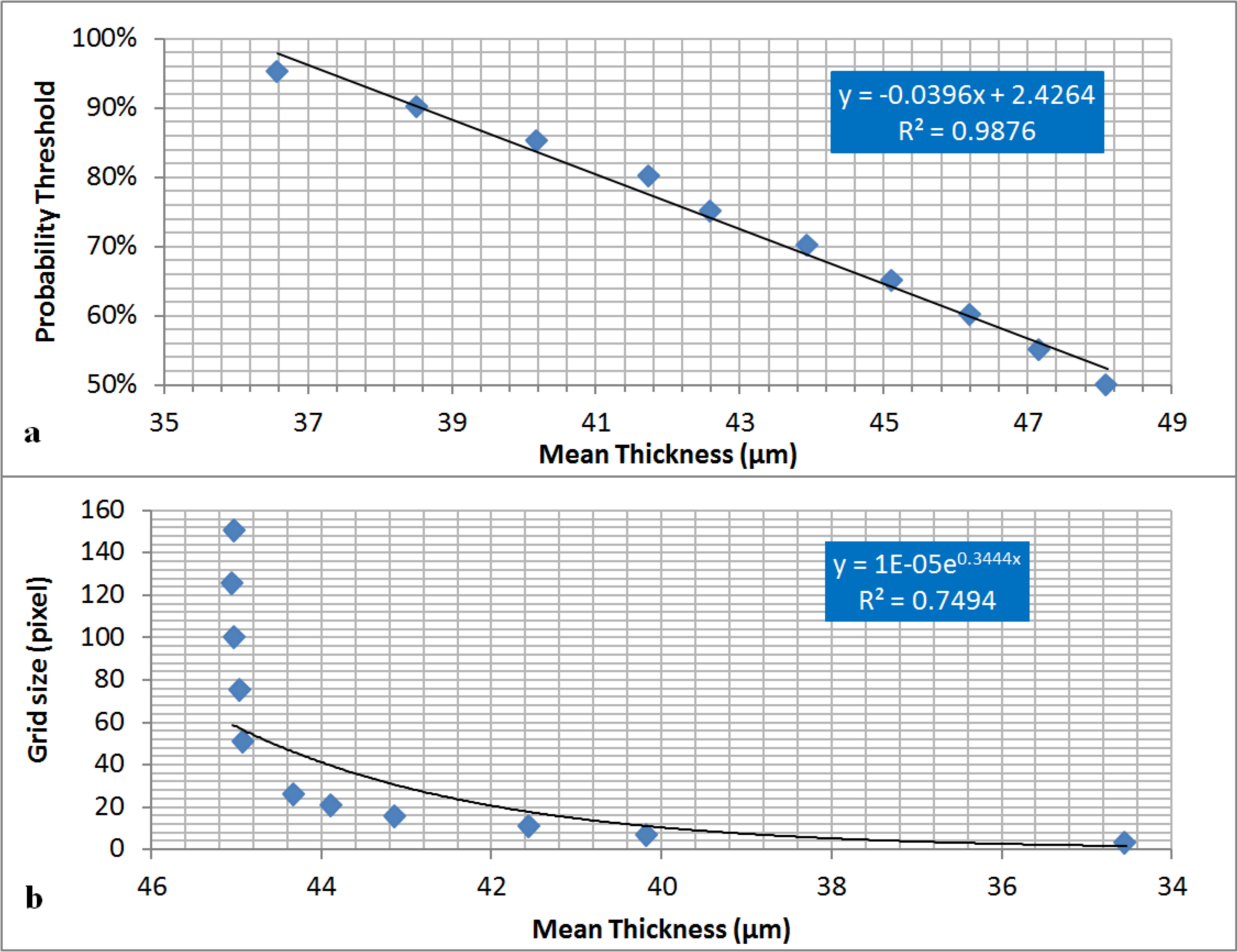
The effect of MIA-Clustering algorithm parameters on average thickness of the wire. (A) Full range of possible probability thresholds and with grid size of 7 held constant. (B) Full range of possible grid sizes with probability threshold held constant at 85%.

**Figure 7:**
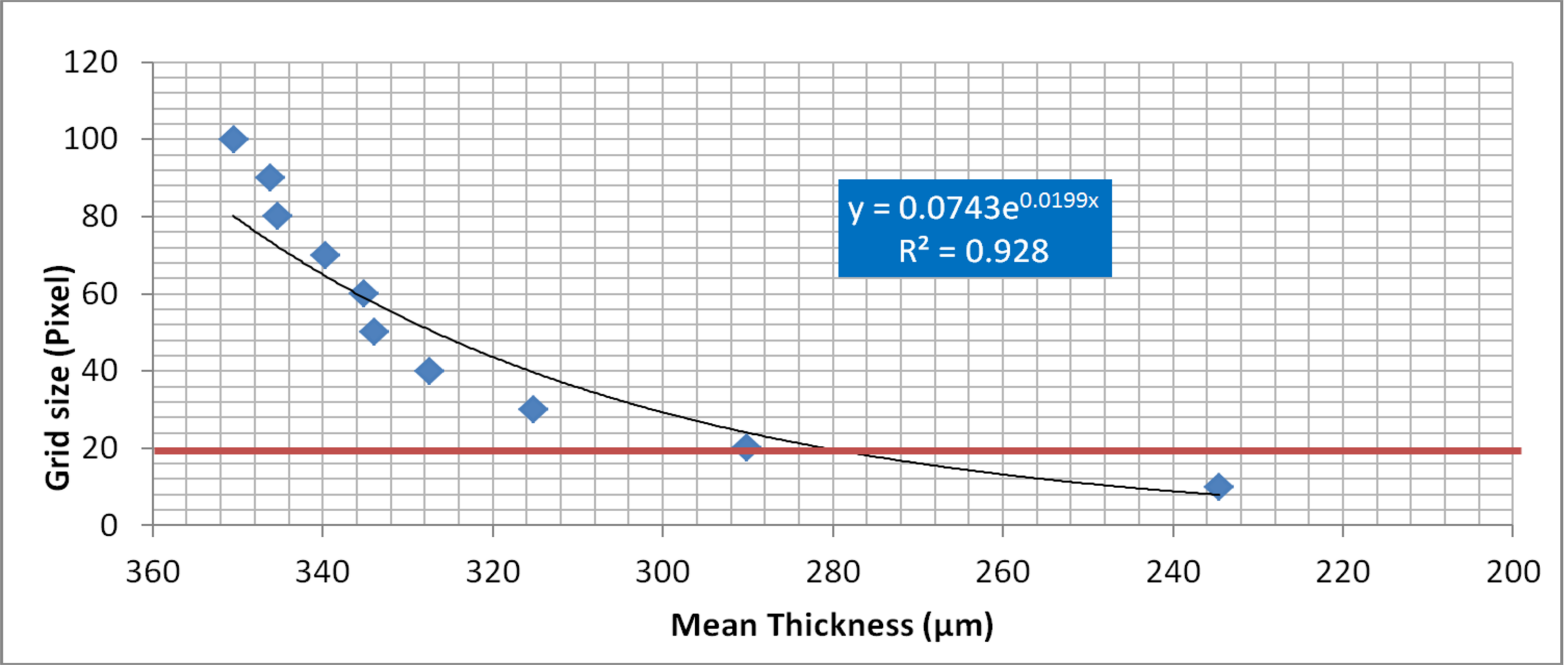
The effect of grid-size input on average thickness estimates of the fossil, after MIA-Clustering segmentation. Grid size ranged from 10 to 100 voxels. The red line represents the grid size of 20, ascertained from manual measurement of the fossil as per the technique in Fig. 2.

In order to evaluate the potential effect of input error in the MIA-Clustering algorithm, the wire was segmented over the full range of each input variable, and average 3D-thickness of each segmentation was measured. Figure 6A demonstrates the linear relationship between probability threshold and thickness for this image. The range of grid-size values result in a thickness range of 12 μm. Figure 6B demonstrates an exponential relationship from the maximum possible (150) to the minimum possible grid size (3) and a thickness range of 9 μm. This parameter quickly converges on values within 10% of the known thickness of the wire when grid size becomes small enough to segment the finer structures of the image at ∼25 voxels. From this point lower grid sizes produce a larger variation in thickness values as fine structures are more consistently segmented, only underestimating thickness when a grid size smaller than the width of the fine structures is used. As expected, different grid sizes produced a wider range of mean thickness measures (∼100 μm) for the structurally variable fossil, than the machined wire (Fig. 7). It should be noted that these values include cortical bone and reflect variation in segmentation of the whole image rather than a trabecular analysis. Despite this larger range, thickness values display an exponential relationship with grid size quickly converging on the value obtained from visual inspection. Much as in the grid size comparison for the machined wire (Fig. 6), when grid size becomes small enough to segment the finer structures of the image at ∼35 voxels variation in thickness increases (Fig. 7). This trend continues until a grid size smaller than the width of the fine structures is used and the method begins to detect inhomogeneities within the osseous structure.

### RCA fossil segmentation

**Figure 8:**
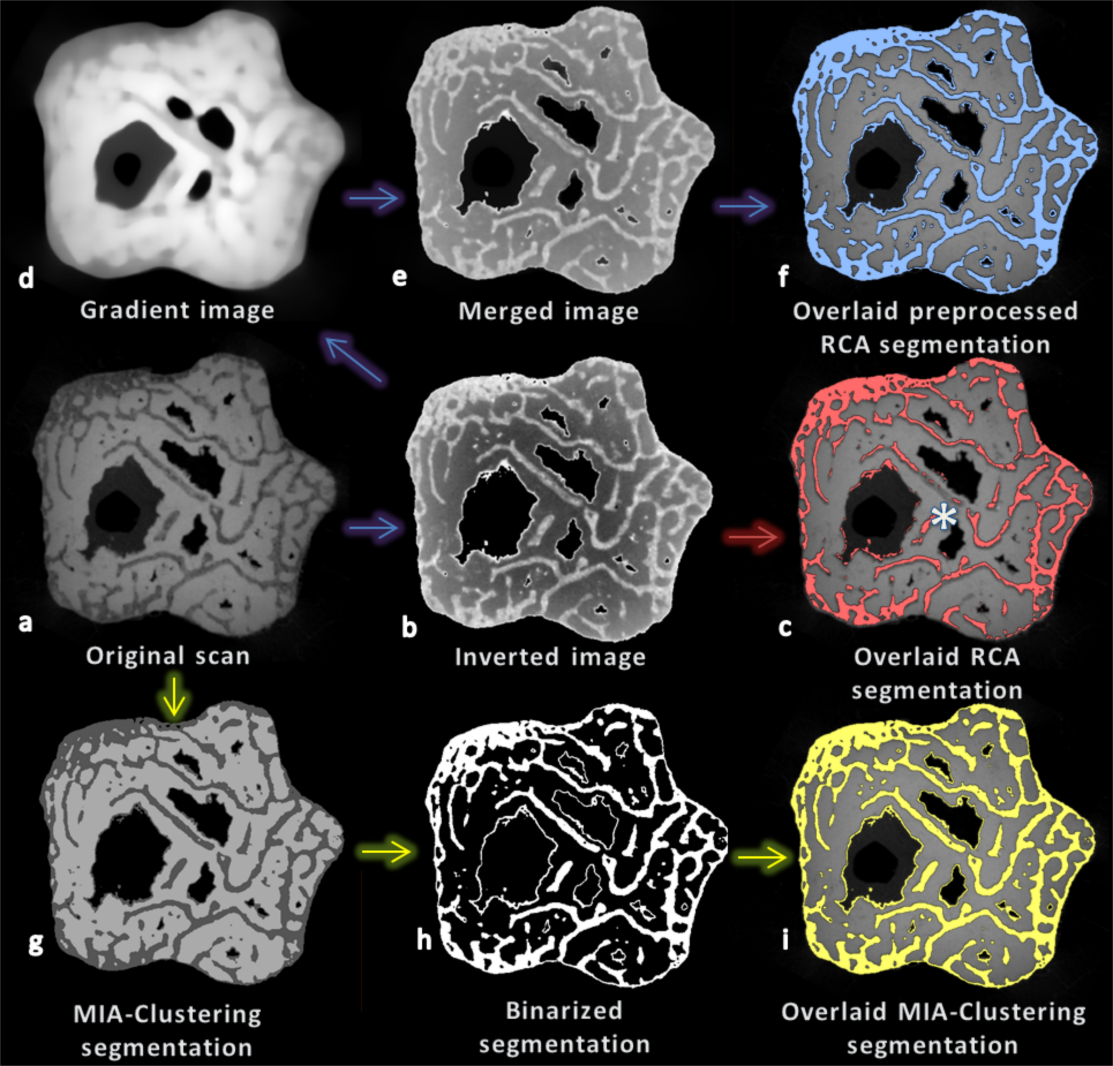
Cross-section (*XY* plane) through the fossil at various stages of segmentation using RCA and MIA-Clustering. (A) The fossil scan. (B) The image after foreground inversion. (C) The RCA segmentation of the inverted image overlaid on the original image (red), note the lack of segmentation of central trabeculae (e.g., above the white asterisk). (D) An image preserving the global gradient of the fossil scan but little of its spatial structure, after a strong median filter. (E) The result of merging the global gradient and the inverted image. (F) The RCA segmentation of the merged result overlaid on the original image (blue). (G) The MIA-Clustering segmentation of the three classes in the image. (H) The MIA-Clustering segmentation binarized on the second brightest class, the fossilized bone phase. (I) This binarized segmentation overlaid on the original image (yellow). See text for further details.

Ray Casting Algorithm is only able to segment the highest attenuation phase in an image, because it will only exclude voxels on the other side of a gradient-defined edge if they have a lower gray value than the phase of interest. Since the structure of interest was not the brightest part the image (Fig. 8A), it was necessary to invert the image in Avizo 6.3 (Visualization Sciences Group, Berlin, Germany, Fig. 8B). A median filter of kernel size three was run as part of the RCA program using a lower threshold of 19,000, an upper threshold of 29,000 and minimum edge strength of 2,500 (Fig. 8C). This was not a satisfactory segmentation of the image, as much of the trabeculae near the center of the bone were lost. Therefore in order to somewhat reduce the artifactual global gradient, the original image was subjected to a median filter of kernel size 25, largely obliterating structures but preserving the global gradient (Fig. 8D). The resultant image could then be added to the inverted image to “cancel-out” the global grayscale gradient without affecting the edge gradients of the trabeculae to a large extent (Fig. 8E). RCA segmentation could then produce an improved segmentation with same parameters as initially used (Fig. 8F).

### MIA-Clustering algorithm fossil segmentation

As a pre-processing step, a noise reducing median filter of kernel size three was applied, and the image was thresholded at 10,000 to remove noise in the background of the image (Fig. 8A). The MIA-Clustering algorithm was run to look for three classes with a grid size of 20, since the thickest elements of the trabecular bone were ∼15 voxels in dimension from a cursory inspection in Avizo 6.3 (Fig. 8G). No probability threshold was needed in this case for refinement, though running the command with a threshold of 50% achieves the same result. Subsequently the image was binarized on the second brightest class in the image, leaving only the fossil bone phase (Fig. 8H). This post-processing step allows for direct comparison with the RCA segmentation but is not necessary (Figs. 8F, 8C and 8I).

## Discussion

### Wire segmentation

The current study presents a novel open-source method for segmenting bone or fossil bone phases from high-resolution μCT images. Tests using a wire phantom indicate that both this technique and RCA are capable of producing accurate segmentations that are within 1% of the wire phantom’s thickness (Table 1; Figs. 4 and 5). Therefore in scans with high material contrast, including those of the present synthetic sample and many examples of dry bone, it appears both segmentation techniques would produce accurate results. However, in practice, the MIA-Clustering algorithm offers several advantages over other segmentation techniques by keeping subjective user decisions to a minimum to increase the reproducibility of results.

### Parameter robusticity

Many segmentation approaches can require manual interaction with the image to provide appropriate input parameters, such as the placement of seed points for a region-based segmentation or the visual inspection of trial RCA segmentations. In this case the user must iteratively determine whether one set of trial RCA parameters produced a better segmentation of the wire phantom than the last and when these parameters could no longer be improved. It can often take many attempts to find acceptable parameters, since there is no objective starting point other than the range of grayscale values in the image for the lower and upper thresholds. Since “minimum edge strength” is not easily visualized, it can be initially difficult to find an acceptable value for this parameter. Conversely, the MIA-Clustering algorithm input parameters are data-led, as grid size selection is based on the dimensions of the structure to be segmented, either through prior knowledge or an initial, manual, inspection of the material (Fig. 2). In the case of the wire, a grid size of seven is just larger than its, known, five voxel thickness and six voxels may be too small due to potential partial volume averaging effects. In the case of the fossil, cursory measurements in three orthogonal 2D-slices of the image were sufficient to determine an appropriate grid size of 20. Average thickness measures of segmentations produced by different grid sizes demonstrate that a grid size of 20 is within the range of values that greatly affect the segmentation result (Fig. 7) but is not so small that algorithm detects inhomogeneities within the phase of interest and begins to break-up and thin trabeculae (Figs. 8G–8I). In both cases, as the grid-size parameter selection was data-led, there was an objective justification for the value used. Though this value may not necessarily produce the optimal MIA-Clustering segmentation, especially in the fossil, it does provide a starting point within a narrow range of values that allow the segmentation of finer structures to varying degrees. Further, as the grid-size parameter defines a local reapplication of a machine-learning algorithm, it could be argued it is more objective than a user-defined threshold of either absolute grayscale values or their gradients. Therefore, this data-led parameter selection requires minimal manual interaction with an image and provides an objective justification for the value used, even when segmenting a structure of largely unknown and variable dimensions, such as osseous or fossil material.

The optional probability threshold parameter, however, is more subjective as it is only found by trialing values. Yet this final step of the algorithm may only fine tune the segmentation from the data-led clustering results. Indeed, over the full range of 50 possible values not only did the segmented wire phantom show just a 30% variation in measured average thickness, it did so in a predictable way with strong a linear relationship (Fig. 6A). This is due to the fact that voxels at the boundary of each segmented phase will have lower membership coefficients than those in the middle on the phase (Fig. 1C). As the threshold is raised, more of these boundary voxels are no longer considered part of this phase and the thickness of the structure will reduce in-kind (Fig. 1F). This relationship allows the user to potentially derive an acceptable value after just two trials. The probability threshold is particularly useful for the accurate segmentation of abrupt phase transitions, such as the edge of the machined wire. In structures with more gradual or complex edge transitions, such as fossilized or extant bone, this parameter is less useful as the effects of different values will be less predictable; the probability threshold was not used in the fossil segmentation. Therefore the MIA-Clustering algorithm keeps subjective user decisions to a minimum by basing input parameters on the properties of the image, rather than iterative manual interaction and more subjective refinement of the result is done in a predictable way, over a small range of input values.

Another way the MIA-Clustering algorithm reduces subjective user decisions is by limiting input parameters to a minimum. The algorithm only takes two input parameters, each with a smaller range of values than the three of RCA, since minimum edge strength ranges from 0 to 32,000 and the thresholding limits are based on the potential gray value range of 16 bit data, 0–65,535. Initially, the relatively small range of inputs for the MIA-Clustering algorithm could be seen as detrimental, affording the researcher less freedom to find values to segment the data accurately. However, this constraint allows for less error in parameter selection and is sufficient to quickly converge on a single pair of parameters that produce an acceptable segmentation (Fig. 6).

An additional benefit to having a small range of input values is that it does not allow for multiple combinations that yield similar results. Here, there are at least two sets of input parameters for the RCA that can produce near identical segmentations and thickness value measurements (Table 1; Figs. 4 and 5). The MIA-Clustering algorithm is not subject to the same equifinality and so results are more reproducible since they can only be achieved via the same input.

The MIA-Clustering algorithm appears to be as accurate as another leading segmentation technique, RCA, in segmenting the wire phantom. Yet the method presented here reduces subjective user decisions to a minimum by grounding input parameters in the properties of the image as well as limiting the range of these input parameters and in doing so, obviating the issue of equifinality. This increased objectivity allows for faster more reproducible segmentations. Indeed, since these parameters are not based on grayscale values but rather the structures at hand, they may be applied uniformly across a sample of different scans of similar synthetic, or dry osseous, material removing another potential source of error in segmentation and measurement across a sample. However, perhaps the most useful property of the MIA-Clustering algorithm is its ability to segment more complex, embedded structures, with less clear contrast, such as fossil material.

### Fossil segmentation

One of the clearest challenges uniquely presented by segmentation of the fossil material is the high-attenuation invasive matrix. As the highest attenuation phase is selected by default in RCA, it was necessary to invert the foreground image, where matrix has a higher attenuation than the fossil bone (Fig. 8B), adding another pre-processing step and a potential source of error. Conversely, the MIA-Clustering method can segment multiple classes at once. Matrix, background and bone may each be a distinct initial cluster set, used to segment the image into separate gray value classes. Any of these classes can be extracted from the image via a simple threshold if subsequent analysis requires a binarized image (Fig. 8H). MIA (Wollny et al., 2013) offers a number of single-task command line tools, including a binarize filter that was used to produce the present result. The highest attenuation structure need not be the one of interest, and so the extra step of inverting the image is not required. Since matrix is also segmented it is also easier to compare the segmentation to the original image by eye, since the white of binarized image may appear larger than the original simply because it is brighter (e.g., Figs. 8A, 8G and 8H).

A further challenge of this particular fossil image is the global gradient which makes the center of the object appear brighter than the edges. The ray casting step of the RCA was invented to close gaps in Sobel filter defined edges that are caused by local grayscale transitions, not steep enough to meet the globally set “minimum edge strength” parameter. The first derivative of grayscale value transitions, rather than absolute values, is still based on a global, if locally applied, threshold. Therefore, although RCA mitigates the effects of a global gradient, it is not immune to them (*contra* Scherf & Tilgner, 2009). The global intensity gradient may affect one side of an edge more than the other if one edge is more central and in doing so, may change the grayscale gradient over the transition. Therefore, RCA may not find edges where they exist in the cases of these artifacts. The present fossil scan appears to be darker in the center of the inverted image (Fig. 8B). RCA accurately segments the trabeculae closer to the edge of the fossil but fails to segment the central trabeculae as their grayscale gradients relative to the matrix phase are not steeper than the “minimum edge strength” threshold applied (Fig. 8C). Ameliorating this global gradient as per the extra pre-processing steps allows the RCA with the same parameters to segment these central trabeculae (Fig. 8F). However, these extra un-prescribed steps make the segmentation process less efficient and potentially less reproducible. The MIA-Clustering algorithm, however, does not use grayscale-based thresholds but considers only the local sub-volumes at the edge or the center of the fossil when segmenting them and can therefore segment the trabeculae in both areas of the bone concurrently (Figs. 8G–8I).

Both fossil segmentations contain thin rings at the boundary of invasive matrix and air as these features are present in the initial image and have similar characteristics as trabecular bone (Figs. 8C, 8F and 8I). While both algorithms fully segment the image, researchers may wish to remove these features, before analysis, as they are not of biological origin. While this is beyond the scope of the current method, we would suggest applying a connected component algorithm, as available in software such as Avizo, to remove many of these features that are unconnected to the segmented bone. Unfortunately, to the authors’ knowledge, remaining connected features must be removed manually at the researcher’s discretion.

Unlike RCA and single threshold methods, the MIA-Clustering algorithm has the flexibility to concurrently segment multiple classes across a fossil specimen affected by a global gradient scanning artifact, segmenting a phase of interest that is not necessarily the brightest in the image. The preservation of multiple classes in the segmentation provides a higher fidelity comparison between the segmentation and the original image. Also, the lack of additional pre-processing steps required for this segmentation allows for fewer potential sources of error and greater reproducibility of results. Therefore, this method is particularly suitable for the segmentation of complex images containing several embedded structures. These images may include fossils with invasive matrix or possibly even images of several tissues produced by magnetic resonance imaging techniques. The presented algorithm can also be used on 8 bit data though the efficacy of the segmentation will depend on the clarity of the original image.

## Conclusion

Here, we present a segmentation algorithm implemented in free open-source software, which can be run on most operating systems and is as effective as other leading algorithms. The move from a gray value-based approach to a data-led, machine-learning approach allows the MIA-Clustering algorithm to lessen the amount of subjective user choices required for segmentation. Therefore, MIA-Clustering segmentations of μCT data offer increased reproducibility. Further, the flexibility of this MIA-Clustering algorithm allows for segmentation of problematic modern or fossil material, which often contains more than two structures and may be affected by common scanning artifacts. The robusticity of the algorithm is demonstrated by the lack of need for additional image processing steps and by how quickly the range of possible input parameters converge on those acceptable for segmentation. The MIA-Clustering algorithm is a flexible, robust method that produces highly reproducible results, ideal for segmenting fossil bone.

## Supplemental Information

10.7717/peerj.4374/supp-1 Supplemental Information 1 Summary of subjective user decisions minimized by the MIA Clustering Algorithm, as discussed in the manuscript text.

10.7717/peerj.4374/supp-2 Supplemental Information 2 Sample of wire (as in Fig. 2). Raw data sample–this is a compressed Nifti (.nii) format volume image.

10.7717/peerj.4374/supp-3 Supplemental Information 3 Sample of fossil (as in Figs. 5 and 6). Raw data sample–this is a compressed Nifti (.nii) format volume image.

## Additional Information and Declarations

### Data Availability

The following information was supplied regarding data availability: The software underpinning this method is freely available online and instructions of how to obtain it from http://mia.sourceforge.net/ are given in the article.
