# Supplementary material for: MIA-Clustering: a novel method for segmentation of paleontological material
Source: PeerJ. 2018 Feb 23;6:e4374. doi: 10.7717/peerj.4374 (PMC5826037; doi:10.7717/peerj.4374)
Supplement: Supplemental Information 1 [file peerj-06-4374-s001.docx]

**Supplementary information 1.**

Summary of subjective user decisions minimized by the MIA Clustering Algorithm, as discussed in the manuscript text.

| **Subjective user-decision** | **Minimized by the MIA-Clustering Algorithm?** | **Minimized by RCA?** |
| --- | --- | --- |
| Initial input parameter selection | Yes, grid-size should be slightly larger than cursory measurement of the structure of interest. | No, while maximum upper and lower thresholds may be found from the data range in the image, appropriate minimum edge strength values are not clear initially. |
| The number of decisions required. | Yes, only the single parameter, grid-size, must be chosen. The additional probability threshold is optional and simply a refinement of the segmentation. | No, three parameters must be chosen, each of which could have a great effect on the segmentation. |
| The input parameter values selected from the possible range. | Yes, grid-size should be slightly larger than the maximum 2D dimension of desired structure and only values close to the initial grid-size value will have a great effect on the segmentation. | No, the full range of 16-bit data grayscale values (0-65,535) and minimum edge strength values from 0-31,000 are available. Further different combinations may produce the same result. |
| How to invert or otherwise, pre-process an image where the desired structure is not the brightest in the image. | Yes, multiple classes can be segmented at once; the desired class can be thresholded out if required. Similarly, other types of data such as 8-bit may be segmented. | No, pre-processing steps required for the algorithm are not standardized and maybe unique to particular images. |
| How to ensure parameters used in segmentation of different scans do not introduce additional variation into the structures analyzed. | Yes, similar structures (such as trabeculae in two fingers of the same hand) should have similar grid-sizes applied. | No, scans of two very similar materials may have different gray values and so require different sets of input parameters. |
